# Reddish-Orange Luminescence from BaF_2_:Eu^3+^ Fluoride Nanocrystals Dispersed in Sol-Gel Materials

**DOI:** 10.3390/ma12223735

**Published:** 2019-11-13

**Authors:** Natalia Pawlik, Barbara Szpikowska-Sroka, Joanna Pisarska, Tomasz Goryczka, Wojciech A. Pisarski

**Affiliations:** 1 Institute of Chemistry, University of Silesia, 40-007 Katowice, Poland; barbara.szpikowska-sroka@us.edu.pl (B.S.-S.); wojciech.pisarski@us.edu.pl (W.A.P.); 2 Institute of Materials Engineering, University of Silesia, 41-500 Chorzów, Poland; tomasz.goryczka@us.edu.pl

**Keywords:** BaF_2_ nanocrystals, glass-ceramics, Eu^3+^ luminescence, sol-gel synthesis

## Abstract

Nanocrystalline transparent BaF_2_:Eu^3+^ glass-ceramic materials emitting reddish-orange light were fabricated using a low-temperature sol-gel method. Several experimental techniques were used to verify structural transformation from precursor xerogels to sol-gel glass-ceramic materials containing fluoride nanocrystals. Thermal degradation of xerogels was analyzed by thermogravimetric analysis (TG) and differential scanning calorimetry method (DSC). The presence of BaF_2_ nanocrystals dispersed in sol-gel materials was confirmed by the X-ray diffraction (XRD) analysis and transmission electron microscopy (TEM). In order to detect structural changes in silica network during annealing process, the infrared spectroscopy (IR-ATR) was carried out. In particular, luminescence spectra of Eu^3+^ and their decays were examined in detail. Some spectroscopic parameters of Eu^3+^ ions in glass-ceramics containing BaF_2_ nanocrystals were determined and compared to the values obtained for precursor xerogels. It was observed, that the intensities of two main red and orange emission bands corresponding to the ^5^D_0_→^7^F_2_ electric-dipole transition (ED) and the ^5^D_0_→^7^F_1_ magnetic-dipole (MD) transition are changed significantly during transformation from xerogels to nanocrystalline BaF_2_:Eu^3+^ glass-ceramic materials. The luminescence decay analysis clearly indicates that the measured lifetime ^5^D_0_ (Eu^3+^) considerably enhanced in nanocrystalline BaF_2_:Eu^3+^ glass-ceramic materials compared to precursor xerogels. The evident changes in luminescence spectra and their decays suggest the successful migration of Eu^3+^ ions from amorphous silica network to low-phonon BaF_2_ nanocrystals.

## 1. Introduction

Among the variety of inorganic amorphous host matrices, mixed oxyfluoride glasses in the presence of barium fluoride BaF_2_ demonstrate a strong potentiality towards the development of near-IR solid-state lasers, broadband fiber amplifiers and highly compact optical devices [1,2,3]. The systematic investigations indicate that several glasses show a better thermal stability, lower phonon energy and weaker OH^−^ absorption coefficient after introduction of BaF_2_ to the base chemical composition. In consequence, it results in larger stimulated emission cross-sections and longer luminescence lifetimes related to near-IR laser transitions of rare earth ions. The influence of glass-modifier BaF_2_ on the positions of luminescence bands of rare earth ions and their relative integrated intensities was examined in detail. The previously published works have been well demonstrated that spectroscopic properties of rare earth ions are changed significantly in tellurite [4], phosphate [5], germanate [6,7,8] and borate [9] glasses, where BaO was substituted by BaF_2_.

Special attention has been paid to silicate glasses modified by BaF_2_ before and after heat treatment. In most cases, the precursor silicate glasses with BaF_2_ were synthesized using the conventional high-temperature melt-quenching method. During heat treatment process of silicate glasses under controlled technological conditions, i.e., annealing time and temperature, fluoride nanocrystals BaF_2_ are quite well formed [10]. Barium fluoride nanocrystals are distributed into glass-host matrices and the received new materials are well-known as transparent glass-ceramics (TGC). For TGC system based on Na_2_O/K_2_O/BaF_2_/Al_2_O_3_/SiO_2_ [11], the mean crystallite sizes of BaF_2_ were in the range from 6 nm to 15 nm and their values increased with time of heat treatment process. Additionally, the increasing of annealing temperature from T = 500 °C to 600 °C resulted in an increase of the crystallite size from 6 nm to 15 nm, respectively. Rare earths as the optically active ions are usually incorporated into fluoride crystalline phase. However, the kind of crystalline phases and rare earths depend critically on technological conditions used to fabricate glass-ceramic materials. The X-ray diffraction analysis of 50SiO_2_-20Al_2_O_3_-20BaF_2_-7NaF-3EuF_3_ based silicate glasses (prepared in reducing atmosphere and then heat-treated at 570 °C, 580 °C and 590 °C [12]) shown prominent diffraction lines of the cubic BaF_2_ nanocrystals with no second crystalline phase. However, besides cubic BaF_2_ fluoride phase, BaAl_2_Si_2_O_8_ crystalline phase could also be observed in the glass sample heat-treated at 600 °C. Further studies indicated that the luminescence spectrum of the precursor silicate glass consisted of a broad blue band assigned to the 5d-4f transition of the divalent Eu^2+^ ions and some sharp orange and red peaks corresponding to the characteristic 4f-4f transitions of trivalent Eu^3+^ ions. It was suggested the co-existence of Eu^2+^/Eu^3+^ ions in precursor glass and concluded that the only part of Eu^3+^ ions have been reduced to Eu^2+^ ions even if the glass was prepared in reducing atmosphere. Completely different results were obtained for glass heat-treated at temperature 570 °C. Thus, almost all trivalent Eu^3+^ ions were reduced to divalent Eu^2+^ ions because only a broad blue emission band corresponding to the 5d-4f transition of the Eu^2+^ could be observed in the glass ceramic containing BaF_2_ nanocrystals [12]. Crystallization processes and optical properties, especially up-conversion and near-infrared luminescence properties, have been examined mainly for transparent silicate glass-ceramic systems containing BaF_2_ nanocrystals and Er^3+^ [13] or Er^3+^/Ln^3+^, where Ln = Yb, Nd [14,15,16]. Differences in the luminescence characteristics of rare earth ions in precursor glasses and transparent glass-ceramics can be explained by structural changes in the local environment around the optically active ions. The spectroscopic consequence of structural transformation from precursor glasses to TGC systems containing BaF_2_ nanocrystals is improvement of luminescent lines of rare earths. The up-conversion and near-infrared luminescence spectra of Er^3+^ ions in transparent glass-ceramics containing BaF_2_ nanocrystals are greatly enhanced in comparison to precursor silicate glasses [13,14,15,16]. The same situation is also well observed for BaF_2_ nanocrystals in silicate TGC systems containing Ho^3+^ or Ho^3+^/Tm^3+^/Yb^3+^ ions, which have been examined for optical amplifiers at 1.2 µm and near-IR lasers around 2 µm [17], and white up-conversion luminescence applications [18].

Transparent glass-ceramics produced by low-temperature sol-gel method are suitable alternative materials for numerous photonic applications. Comprehensive results for glass-ceramic sol-gel materials containing fluoride nanocrystals are presented and discussed in the excellent reviewed paper published recently [19]. In fact, only a few reports is dedicated to transparent glass-ceramics containing divalent MF_2_ (M = Ca, Sr, Ba) fluoride nanocrystals prepared by sol-gel method and characterized by low phonon energy and large transfer coefficient between rare earth ions. The sol-gel fabrication and optical characterization of Er^3+^-doped silicate glass-ceramics with the presence of BaF_2_ nanocrystals was reported for the first time by Chen et al. [20]. In this work, oxyfluoride silicate xerogels containing Eu^3+^ ions have been examined before and after heat treatment. The precursor xerogels were heat-treated in order to obtain transparent glass-ceramics containing BaF_2_ crystalline phase at nanometric scale. In particular, luminescence spectra of Eu^3+^ ions and their decays measured in TGC samples have been investigated and compared to precursor silicate xerogels.

## 2. Materials and Methods

All of the reagents used during sol-gel preparation procedure were of analytical purity from Aldrich Chemical Company and used without further purification. Deionized water was taken from Elix 3 system (Millipore, Molsheim, France).

The Eu^3+^-doped precursor xerogel samples with nominal composition (in molar ratio): TEOS:C_2_H_5_OH:H_2_O:CH_3_COOH = 1:4:10:0.5 (90 wt. %) CF_3_COOH:Ba(CH_3_COO)_2_:Eu(CH_3_COO)_3_ = 5:1:0.05 (10 wt. %) were synthesized. In the presented procedure the mixtures of tetraethoxysilane (TEOS), ethanol, water and acetic acid were put into round-bottom flasks and stirred for 30 min to perform the hydrolysis reaction. Simultaneously, Ba(CH_3_COO)_2_ and Eu(CH_3_COO)_3_ were dissolved in water and trifluoroacetic acid (TFA). In the next step, the obtained solutions were introduced into hydrolyzed tetraethoxysilane (TEOS) and mixed for another 60 min. Afterwards, the sols were dried at 35 °C for 7 weeks to form transparent and colorless xerogels. To fabricate the glass-ceramics containing BaF_2_ nanocrystals, the xerogels were annealed in a muffle furnace FCF 5 5SHP (Czylok, Jastrzębie-Zdrój, Poland) at 350 °C. The temperature was raised by 10 °C /min until 350 °C was achieved and the samples were annealed for 10 h. After this time, resulted glass-ceramic materials were cooled down to room temperature in a closed furnace.

The samples were characterized by a SETARAM Labsys thermal analyzer (SETARAM Instrumentation, Caluire, France) using the TG and DSC method. The DSC curves were acquired with heating rate of 10 °C /min and the curves (TG/DSC) were registered in a temperature range from 40 °C to 500 °C. In order to examine the structural properties of prepared silica sol-gel samples the IR-ATR spectra were performed on the Nicolet iS50 ATR spectrometer (Thermo Fisher Scientific, Waltham, MA, USA) in the frequency region 500–4000 cm^−1^. To verify the nature of prepared samples and to identify the crystal phase, the X-ray diffraction analysis was carried out using an X’Pert Pro diffractometer supplied by PANalytical (Almelo, Netherlands) with CuKα radiation. Microstructure was observed using JEOL JEM 3010 electron transmission microscope (JEOL, Tokyo, Japan) operated at 300 kV. To supplement, the EDS analysis was also carried out using JEOL microscope. To examine the optical behavior of prepared sol-gel materials, the luminescence measurements were performed using a Horiba Jobin-Yvon FluoroMax-4 spectrofluorimeter (Horiba Jobin Yvon, Longjumeau, France) with 150 W xenon lamp as a light source. The spectral resolution was ±0.1 nm. Decay curves were detected with the accuracy of ±2 μs. All structural and photoluminescence measurements were performed at room temperature.

## 3. Results and Discussion

### 3.1. Structural and Thermal Investigations of Fabricated Silicate Xerogels

**Figure 1:**
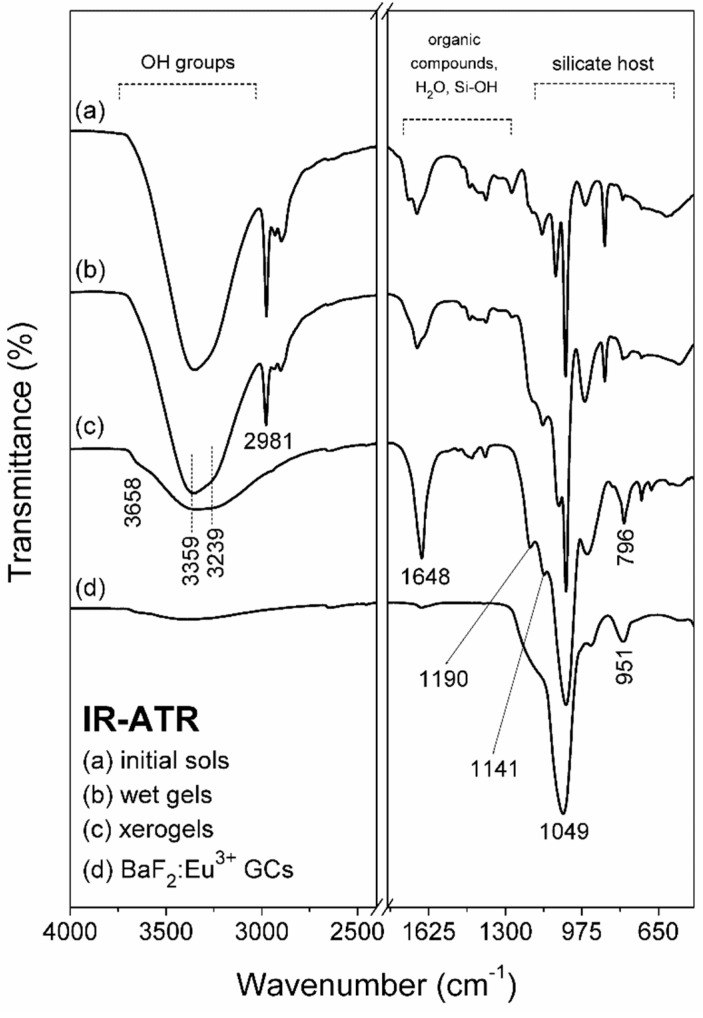
IR-ATR spectra recorded during performed sol-gel synthesis.

To monitor the structural differences in fabricated initial sols, wet-gels and xerogels, the IR-ATR spectra were recorded in the frequency region from 500 cm^−1^ to 4000 cm^−1^. The individual infrared signals were identified based on following papers [21,22]. The *initial sols* (Figure 1a) obtained directly after synthesis revealed the impressive amounts of hydrogen-bonded Si-OH groups (~3359 cm^−1^) originated from hydrolyzed tetraethoxysilane. Moreover, the IR signal located near ~1648 cm^−1^ frequency region could be also assigned to vibrations within Si-OH moieties. Based on infrared signals originated from Si-O-Si bridges (~1190 cm^−1^, ~796 cm^−1^), SiO_4_ tetrahedrons within Q^4^ (~1141 cm^−1^), Q^3^ (~1049 cm^−1^) as well as Q^2^ units (~951 cm^−1^), we concluded that the polycondensation reaction has been started at early stage of sol-gel transformation. The *initial sols* were particularly rich in C_2_H_5_OH, CH_3_COOH catalyst, unreacted TFA residues and water, what could be proven by strong infrared signals recorded from OH groups: ~3239 cm^−1^, C-H vibrations: ~2981 cm^−1^, ~2930 cm^−1^, ~2895 cm^−1^, C=O: ~1648 cm^−1^. The signals assigned to C-F vibrations inside Ba(CF_3_COO)_2_ and Eu(CF_3_COO)_3_ were also identified (near ~1190 cm^−1^ and ~1141 cm^−1^). It was observed that after one week of drying at 35 °C the *initial sols* were transformed into *wet-gels* (Figure 1b). The gelation process resulted in the formation of a liquid-filled highly porous silicate network. The indicated step of sol-gel evolution was related with gradual evaporation of water molecules and organic compounds from microporous silicate structure, which was still dynamic. Indeed, the broad OH-band (~3239 cm^−1^) began to decrease, similarly as in the case of C-H signals (~2981 cm^−1^, ~2930 cm^−1^, ~2895 cm^−1^). It is quite interesting that the amounts of water and organic solvents were still large at indicated transformation step. Such a phenomenon could be explained by low drying temperature (35 °C) and probable possibility for their successfully ‘trapping’ inside microporous silicate network due to strong hydrogen-bonding with unreacted Si-OH groups (~3358 cm^−1^). Simultaneously, the amounts of hydrogen-bonded Si-OH groups gradually decreased what confirmed that the polycondensation reaction was still in progress. In next six weeks, the collapse of the silicate network occurred as a result of compressive stress imposed by capillary forces of the drying liquids. In sum, we observed that the further reaction between unreacted Si-OH groups was promoted. Hence, the growing number of Si-O-Si siloxane bridges (~1190 cm^−1^, ~796 cm^−1^) reinforced the silicate network. For *xerogels* obtained after seven weeks from synthesis (Figure 1c), we identified the presence of vicinal or geminal Si-OH groups (~3658cm^−1^), hydrogen-bonded Si-OH moieties (~3359 cm^−1^) as well as hydrogen-bonded OH groups (~3239 cm^−1^) from residual water and organic solvents. The presence of Si-O-Si siloxane bridges (~1190 cm^−1^, ~796 cm^−1^) and linkages within Q^n^ units (n = 4: ~1141 cm^−1^, n = 3: ~1049 cm^−1^, n = 2: ~951 cm^−1^) inside formed three-dimensional silicate network was also confirmed. Moreover, the infrared signals originated from trifluoroacetates (~1190 cm^−1^ and ~1141 cm^−1^) were also identified. It was also observed that during subsequent steps of sol-gel transformation (*initial sols*, *wet gels* and *xerogels*) the intensity of peak located near ~1648 cm^−1^ frequency region, assigned to vibrations of C=O and Si-OH groups, increased. We suppose that it resulted from densification of sol-gel materials during drying at 35 °C.

**Figure 2:**
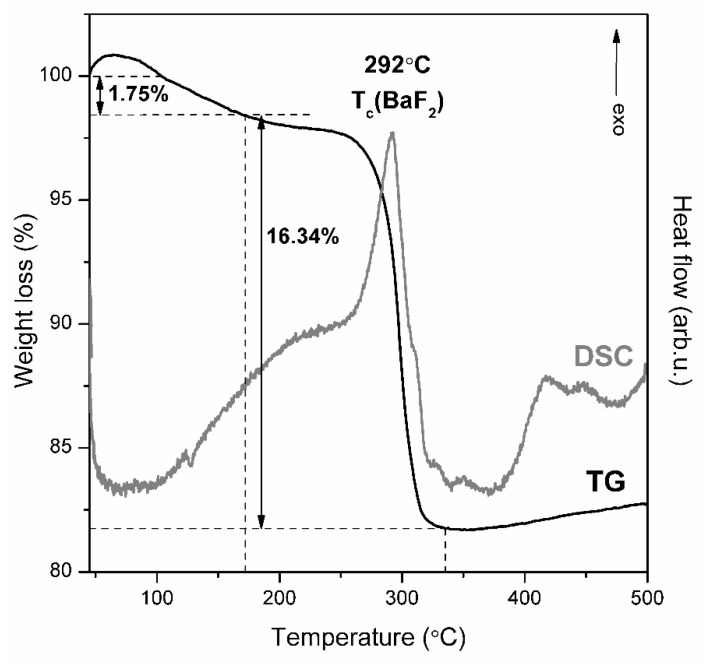
TG/DSC curves recorded for prepared precursor silicate xerogels.

To evaluate the conditions of controlled heat-treatment to transform of fabricated xerogels into glass-ceramics, the TG/DSC analysis was carried out and the recorded curves were depicted in Figure 2. It was recorded the two-stage thermal degradation profile: the first stage was recorded in range from 40 °C to 172 °C and the second stage was recorded in range from 172 °C up to 321 °C. The first stage was registered as a gentle degradation and the resultant weight-loss was estimated to 1.75%. Such degradation step is related with evaporation of residual volatile components (water, ethanol, acetic acid, exceed of TFA acid) from porous silicate network. The remaining TFA acid reacted with acetate salts introduced during initial step of sol-gel synthesis and–in consequence of chemical reaction between them–the trifluoroacetates were obtained as products. During further rise in temperature, a strong exothermic DSC peak with maximum located at 292 °C was recorded and simultaneously, the huge weight loss was also observed (16.34%). Hence, the second degradation step was ascribed to thermal degradation of Ba(CF_3_COO)_2_ and formation of BaF_2_ crystal phase. Based on presented results, the 350 °C temperature was chosen to carry out the controlled ceramization process of precursor xerogels due to two reasons. Firstly, the amount of introduced Ba(CH_3_COO)_2_ during synthesis was relatively small (3.0 wt. %) and therefore, the heat-treatment performed at 350 °C ensured the crystallization process. Secondly, the resultant silicate sol-gel hosts seems to be thermally stable at 350 °C and the further rise in temperature did not caused significant changes in masses of fabricated xerogels.

The IR signals recorded after controlled heat-treatment (Figure 1d) were mainly assigned to the vibrational modes characteristic to the silicate network. It was observed that band arising from residual organic solvents and water disappeared due to their evaporation from pores during controlled heat-treatment process. It should be also noted that performed heat-treatment process was accompanied with polycondensation reaction of silicate network, hence, Si-OH groups reacted to each other and formed Si-O-Si siloxane bridges. Indeed, the maximum of such broad OH-band was shifted to ~3400 cm^−1^, what indicated the presence of Si-OH hydrogen-bonded groups, however, the weak intensity of indicated band suggests their small amounts within sol-gel network. The weak IR peak located at ~1648 cm^−1^ frequency region was also detected, which was mainly coming from such residual Si-OH moieties (indicated IR signal should not originated from C=O groups due to evaporation of organic compounds and thermal decomposition of trifluoroacetates). Next, the shoulder located at ~1190 cm^−1^ and signal at ~796 cm^−1^ were attributed to Si-O-Si bridges created within silica sol-gel network. Finally, the intense IR signal located at ~1049 cm^−1^ was detected and originated from SiO_4_ tetrahedrons inside Q^3^ units. The indicated changes in recorded IR-ATR spectra clearly pointed to evaporation of organic components and water molecules as well as to densification of silicate network during proposed heat-treatment of precursor xerogels. Moreover, it was also observed – what is particularly important from fabrication of fluoride crystals point of view–that shoulders originated from C-F vibrations (~1190 cm^−1^, 1141 cm^−1^) also disappeared.

**Figure 3:**
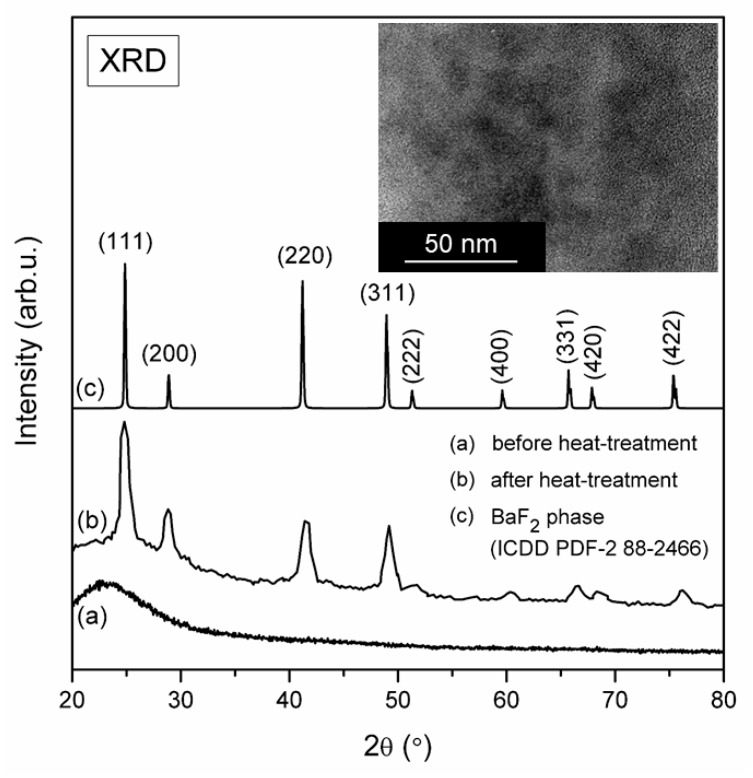
X-ray diffraction patterns for fabricated sol-gel samples: silicate xerogel and BaF_2_:Eu^3+^ glass-ceramic obtained after controlled heat-treatment at 350 °C. Inset shows TEM image of BaF_2_:Eu^3+^.

To verify the nature of prepared sol-gel samples both before and after controlled ceramization process, the X-ray diffraction measurements were carried out (Figure 3). For xerogels, a broad halo pattern characteristic for amorphous system without long-range order was recorded. The controlled heat-treatment process conducted at 350 °C was accompanied by the appearance of diffraction lines, which indicated a crystallization of fluoride phase. Indeed, the XRD patterns are in accordance with the standard diffraction lines of regular BaF_2_ phase from ICDD (The International Centre for Diffraction Data, PDF-2 No. 88-2466) crystallized in *Fm3m* space group. The broadening of recorded diffraction lines indicates that BaF_2_ phase crystallizes in nanometric range. The average crystals size was estimated using the Scherrer Equation (1):

(1) $$D=\frac{K\lambda }{\beta \cos\theta }$$

where D is the crystal size, K is a constant value (for our calculations it was taken K = 1), λ is the X-ray wavelength, β is a half width of the diffraction peak and θ is the diffraction angle. The mean value of BaF_2_ nanocrystals was equaled to 10.8 nm.

It was observed a slight shift of some diffraction lines, which is related with substitution of Ba^2+^ ions in BaF_2_ lattice by Eu^3+^ cations with different ionic radii (Eu^3+^: 1.07 Å [23], Ba^2+^: 1.35 Å [24]). Since the ionic radius of Eu^3+^ ion is smaller than Ba^2+^, it was observed a slight shift of diffraction lines towards higher angle. The inset of Figure 2 presents TEM image of fabricated glass-ceramics. The size of BaF_2_ nanocrystals is consistent with average crystal size estimated from Scherrer equation. To compare, the annealing conditions and sizes of BaF_2_:Eu^3+^ nanocrystals in another glass-ceramics presented in literature were shown in Table 1 [25,26,27,28,29]. The similar crystal size was identified for sol-gel glass-ceramic materials (95SiO_2_-5BaF_2_): 1% Eu^3+^ (mol %) obtained during controlled heat-treatment at 800 °C per 1 h [25] as well as for conventional glasses with composition of 68SiO_2_-15BaF_2_-13K_2_CO_3_-2.75La_2_O_3_-1Sb_2_O_3_-0.25Eu_2_O_3_ (mol %) during annealing at 600 °C per 24 h (6–10 nm) [26]. Additionally, to determine the distribution of individual chemical elements, we performed the analysis from selected sample area containing BaF_2_ nanocrystal using energy dispersive X-ray spectroscopy, EDS. The content of Ba (14 wt. %) and F (10 wt. %) (BaF_2_ nanocrystal), as well as Si (40 wt. %) and O (36 wt. %) (silicate sol-gel host), were easy to determine. However, since Eu^3+^ ions were introduced into sol-gel hosts as dopant, their concentration was below the quantification limit.

According to work by Brown et al. [30], Eu^3+^ ions are located in *C_3v_* symmetry sites in BaF_2_ crystal lattice, despite Ba^2+^ cations are in *O_h_* symmetry sites. Such effect was explained by charge compensation if divalent Ba^2+^ ion in BaF_2_ nanocrystal is substituted by trivalent Eu^3+^ dopant ions, which induces the localization of F^-^ anions in interstitial position or creation of cation vacancies. The detailed mechanisms of charge compensation when Eu^3+^ ions substitute M^2+^ cation in MF_2_ crystal lattice was performed in excellent work by Pan et al. [31].

### 3.2. Photoluminescence of Eu^3+^ in Xerogels and Glass-Ceramics Containing BaF_2_ Nanocrystals

**Figure 4:**
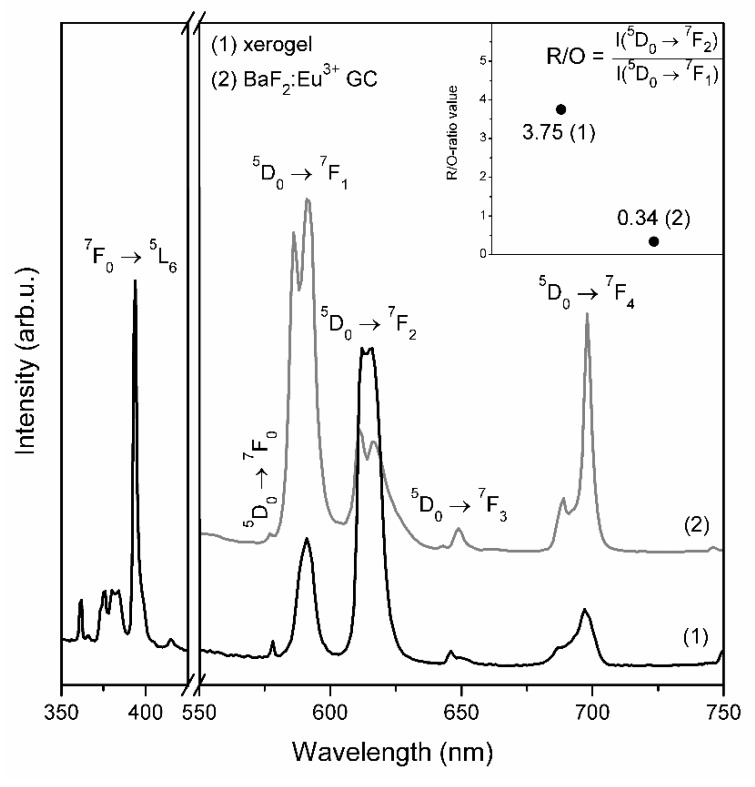
PLE and PL spectra recorded for prepared xerogels and BaF_2_:Eu^3+^ glass-ceramics.

Figure 4 presents the photoluminescence excitation PLE spectra for synthesized silicate xerogels. The spectra were recorded from 350 nm to 550 nm spectral range and monitored at λ_em_ = 611 nm red emission wavelength (the ^5^D_0_→^7^F_2_ transition of Eu^3+^ ions). For fabricated samples, the most prominent line corresponds to the ^7^F_0_→^5^L_6_ transition and thus, the appropriate wavelengths was used to carry out the emission measurements.

As was also demonstrated in Figure 4 (photoluminescence PL spectra) for xerogels, the characteristic bands of Eu^3+^ ions corresponded to the intra-configurational ^5^D_0_→^7^F_J_ transitions within 4f^6^ manifold were recorded: 578 nm (J = 0), 591 nm (J = 1), 612 nm/615 nm (J = 2), 646 nm (J = 3), 697 nm (J = 4). It was clearly observed that for fabricated xerogels the red emission line assigned to the ^5^D_0_→^7^F_2_ electric-dipole transition is more intense compared to the ^5^D_0_→^7^F_1_ orange band. The latter one is a magnetic-dipole transition in nature, which intensity is rather independent of the host. Conversely, the ^5^D_0_→^7^F_2_ is known as a hypersensitive transition and it is easily affected by the local vicinity around Eu^3+^ ion. Hence, the ratio between the ^5^D_0_→^7^F_2_ (R) and the ^5^D_0_→^7^F_1_ (O) emission intensities (R/O) can be considered as a valuable tool for estimation the symmetry in local surrounding around Eu^3+^ ions. The R/O-ratio value calculated for prepared xerogels was estimated to 3.75.

The emission spectrum registered for glass-ceramics containing BaF_2_ nanocrystals obtained after controlled heat-treatment at 350 *°*C revealed some splitting of the ^5^D_0_→^7^F_J_ luminescence lines: 586 nm/591 nm (J = 1), 611 nm/616 nm (J = 2) and 689 nm/698 nm (J = 4). The ^5^D_0_→^7^F_0_ and the ^5^D_0_→^7^F_3_ bands remained unsplit and the maxima of individual lines were detected at 577 nm as well as at 649 nm, respectively. According to excellent paper concerning on spectroscopy of trivalent europium ions by Binnemans [32], the ^7^F_J_ energy levels of Eu^3+^ ions in crystal lattice split in adequate number of sublevels depending on the site symmetry and the J number. Therefore, if Eu^3+^ ion is located in C_3v_ site in BaF_2_ crystal lattice, the J term of the ^7^F_J_ levels should split into two, three, five and six components for the ^5^D_0_→^7^F_1_, ^5^D_0_→^7^F_2_, ^5^D_0_→^7^F_3_ and ^5^D_0_→^7^F_4_ transitions, respectively. Taking into account that Eu^3+^ ions are distributed between BaF_2_ nanocrystals and amorphous silicate sol-gel hosts, such strong split has not been observed. Moreover, a significant growing in intensity of the orange ^5^D_0_→^7^F_1_ band was observed for fabricated BaF_2_:Eu^3+^ GC and the R/O-ratio value was estimated to 0.34. Therefore, it was observed 11-fold decline in R/O-ratios (from 3.75 to 0.34). Such changes in emission profile of Eu^3+^ ions in fabricated glass-ceramics clearly points to change the symmetry in nearest vicinity around dopant ions and nature of bonding character between Eu^3+^ and their framework from covalent to more ionic [30,33,34]. Thus, we could suggest the partial entering of optically active Eu^3+^ ions into precipitated BaF_2_ nanocrystals produced during controlled heat-treatment. To compare, the 2.1-fold (from 1.8 to 0.86) and 3-fold (from 1.8 to 0.6) decline in R/O-ratio value were described for (95SiO_2_-5BaF_2_):1%Eu^3+^ (mol %) sol-gel glass-ceramics fabricated during controlled ceramization performed at 320 *°*C and 800 *°*C, respectively [25].

**Figure 5:**
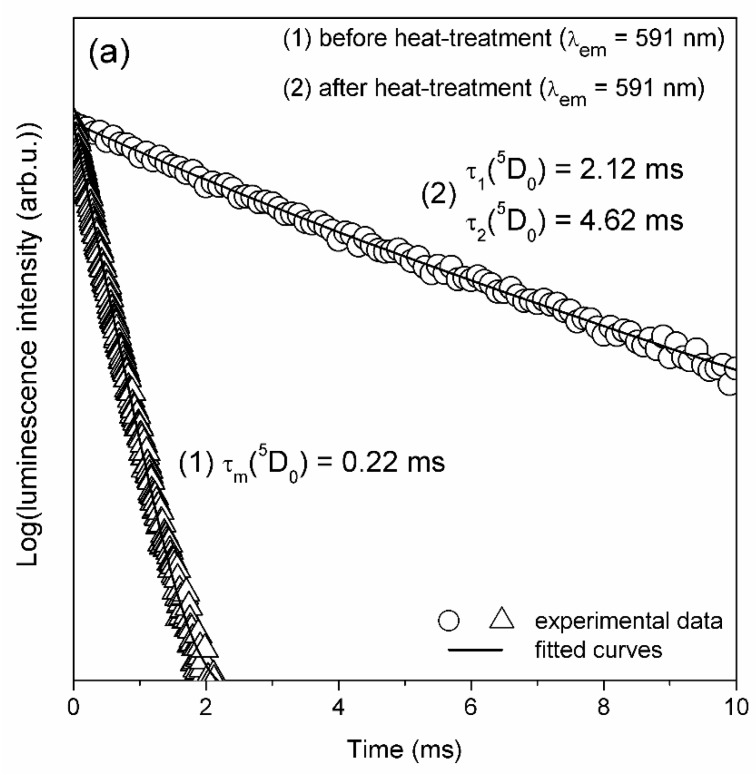
Luminescence decay curves for the ^5^D_0_ excited state of Eu^3+^ ions recorded for orange emission line (λ_em_ = 591 nm) under near-UV illumination.

The photoluminescence decay curves registered for studied sol-gel materials before and after controlled ceramization were shown in Figure 5. Luminescence lifetimes of the ^5^D_0_ excited state of Eu^3+^ ions were measured by monitoring orange emission related to the ^5^D_0_→^7^F_1_ optical transition. The decay curve registered for precursor xerogels is well-fitted to monoexponential function and the estimated luminescence lifetime is equal to τ = 0.22 ms. Relatively short luminescence lifetime is an effect of high-vibrational OH groups neighborhood in local surrounding of Eu^3+^ ions. To cover an energy gap of Eu^3+^ ions between the ^5^D_0_ and the ^7^F_6_ energy states (ΔE = 12500 cm^−1^), only about four OH phonons are required. Therefore, the probability of the ^5^D_0_ luminescence quenching is relatively high. The luminescence decay curve recorded for BaF_2_:Eu^3+^ glass-ceramic samples is well-fitted to bi-exponential function. Calculated luminescence lifetimes are equal to τ_1_ = 2.12 ms and τ_2_ = 4.62 ms. The bi-exponential character of decay curves clearly indicates that two decay channels are involved in the decay process. Therefore, we suppose that optically active dopant ions could be distributed between two different surroundings, i.e., silicate amorphous sol-gel network and BaF_2_ nanocrystals. The shorter luminescence lifetime (τ_1_) is attributed to Eu^3+^ ions surrounded by silicate solgel network (1049cm^−1^ from Q^3^ units of SiO_4_ tetrahedrons, as was evidenced by infrared measurements presented in Figure 1). It is quite interesting that calculated shorter lifetime component (τ_1_) is elongated compared to lifetime value before ceramization. It could be explained by removal of high-vibrational OH groups from water molecules and ethyl alcohol with vibrational energy about 3239 cm^−1^. A longer lifetime component (τ_2_) could be related to location of Eu^3+^ ions in low-vibrational chemical surrounding. In this case, up to ~39 phonons of BaF_2_ crystal lattice (with phonon energy equals to 319 cm^−1^ [35]) are required to cover the energy gap between the ^5^D_0_ and the ^7^F_6_ states of Eu^3+^ ions. Such low-phonon energy environment strongly promotes the radiative relaxation and therefore, the photoluminescence from the ^5^D_0_ level is long-lived. The similar, average lifetime equaled to 4.70 ms ± 0.02 ms was determined by Secu et al. for (95SiO_2_-5BaF_2_):1%Eu^3+^ (mol %) glass-ceramic system (for red luminescence, λ_em_ = 620 nm) after controlled heat-treatment carried out at 350 *°*C.

From the potential applications point of view, this is very important to determine of photoluminescence quantum yield. The systematic investigations clearly indicate that quantum yield can be quite well derived from the luminescence spectra of Eu^3+^. The ratio of the radiative transition probabilities A_RAD_ of the ^5^D_0_→^7^F_J_ (where J = 2, 4) electric-dipole transitions and the ^5^D_0_→^7^F_1_ magnetic-dipole transition of Eu^3+^ in terms of the ratio of areas S under corresponding luminescence bands can be estimated using the following Equation (2) [36]:

(2) ARAD(D50→F72,4)ARAD(D50→F71)=S(D50→F72,4)S(D50→F71)

The ^5^D_0_→^7^F_1_ magnetic-dipole transition of Eu^3+^ plays the role as an internal reference, because it is largely unaffected by the crystal field. For sol-gel systems, the radiative transition probability A_RAD_ (^5^D_0_→^7^F_1_) is equal to 51.9 s^−1^ [37]. The radiative transition probabilities A_RAD_ of the ^5^D_0_→^7^F_J_ (J = 2, 4) electric-dipole transitions calculated for precursor xerogel and sol-gel glass-ceramic containing BaF_2_:Eu^3+^ nanocrystals are close to 204.8 s^−1^ and 35.3 s^−1^, respectively. Finally, the total A_RAD_ values were obtained by summing over the radiative transition probabilities for each ^5^D_0_→^7^F_J_ (J = 1, 2, 4) magnetic-dipole and electric-dipole transitions of Eu^3+^. Thus, the A_RAD_ values for xerogel and glass-ceramic with BaF_2_:Eu^3+^ nanocrystals seems to be 256.7 s^−1^ and 87.2 s^−1^. Then, these values were used to calculate photoluminescence quantum yield expressed by Equation (3):

(3) $$\eta =\frac{A_{\mathrm{RAD}}}{A_{\mathrm{RAD}}+A_{\mathrm{NRAD}}}$$

The sum of radiative (A_RAD_) and non-radiative (A_NRAD_) transition probabilities correspond to the inverse of luminescence lifetime (1/τ) obtained from decay curve measurements. For glass-ceramic with BaF_2_ nanocrystals the luminescence decay curve for the ^5^D_0_ (Eu^3+^) state is bi-exponential with two components: faster (τ_1_ = 2.12 ms) and slower (τ_2_ = 4.62 ms) (Figure 5). Thus, the average lifetime τ_avg_ of ^5^D_0_ (Eu^3+^) can be evaluated using Equation (4) [38]:

(4) $$\tau_{\mathrm{avg}}=\frac{A_{1}\tau_{1}^{2}+A_{2}\tau_{2}^{2}}{A_{1}\tau_{1}+A_{2}\tau_{2}}$$

where A_1_ and A_2_ are fitting constants close to 4.18232 × 10^6^ and 2.51746 × 10^6^, respectively. The τ_avg_ value equals to 4.08 ms. The photoluminescence quantum yield is changed drastically from 6% to 35.6% during transformation from precursor xerogel to glass-ceramic containing BaF_2_:Eu^3+^ nanocrystals. The QY value for glass-ceramic sample with BaF_2_:Eu^3+^ nanocrystals is similar to the results obtained previously for other silicate host lattices such as CaSiO_3_:Eu^3+^ (η~33%) [39], Zn_2_SiO_4_:Eu^3+^ (η~30%) [40] and the commercial red-emitting Y_2_O_2_S:Eu^3+^ (η = 35%) [41,42]. It is also consistent with the results (η = 29/35%) for Eu^3+^ doped CaF_2_, SrF_2_ and BaF_2_ particles synthesized via the fluorolytic sol–gel route [35]. However, direct comparison with other Eu^3+^-doped particles is difficult because the quantum yield depends on several factors like particle size. In general, the optical behavior and several spectroscopic parameters of rare earths like the quantum yield are affected by the refractive index of the host matrix (the surrounding medium), the particle size, the size distribution and the shape of the particles [43].

It is also interesting to notice that the quantum yields for xerogel and sol-gel glass-ceramic with BaF_2_:Eu^3+^ nanocrystals are in a quite good agreement with the results obtained using an alternative method given below. The intrinsic quantum efficiency can be calculated according to relation Φ_Eu_ = k_R_/k, where k corresponds to the total radiative transition probabilities A_RAD_ = 1/τ determined from luminescence lifetime measurements and k_R_ given by Equation (5) [44]:

(5) $$k_{R}=A_{\mathrm{MD},0}n^{3}\left(\frac{I_{\mathrm{tot}}}{I_{\mathrm{MD}}}\right)$$

where I_tot_ and I_MD_ are the integrated emission intensities corresponding to the total ^5^D_0_→^7^F_J_ transitions and the ^5^D_0_→^7^F_1_ magnetic-dipole transition, respectively. In this relation, A_MD,0_ denotes the Einstein spontaneous emission coefficient for the ^5^D_0_→^7^F_1_ magnetic-dipole transition (in vacuum) and its value is close to 14.65 s^−1^ [45], whereas n is the refractive index of the medium. For sol-gel oxyfluoride sol-gel silica systems [46], the refractive index is changed slightly from 1.50 (sample heat-treated at 350 °C) to 1.54 (sample heat-treated at 750 °C) and its value is nearly the same compared to cubic BaF_2_ (1.47–1.48). Thus, BaF_2_ particles can be matched exactly with a glass, xerogel or polymer matrix [47]. Considering Equation (4) the intrinsic quantum efficiency increases from 5.8% (xerogel) to 33% (sol-gel glass-ceramic with BaF_2_:Eu^3+^ nanocrystals). It suggests that oxyfluoride sol-gel glass-ceramics containing BaF_2_:Eu^3+^ nanocrystals are quite good candidates for tunable reddish-orange emitting sources.

## 4. Conclusions

Transparent glass-ceramic materials containing BaF_2_ fluoride nanocrystals were fabricated by low-temperature sol-gel method and then examined using several experimental techniques: TG/DSC, XRD, TEM, EDS, IR-ATR and luminescence spectroscopy. Thermal decomposition of Ba(CF_3_COO)_2_ was identified using TG/DSC measurements, whereas the structural changes in sol-gel silica network were verified by the IR-ATR spectroscopy. The presence of BaF_2_ nanocrystals was confirmed by the XRD measurements and TEM microscopy. The average nanocrystal size was estimated using the Scherrer formula and its value is equal to 10.8 nm, which was also confirmed from TEM image. The enhanced reddish-orange luminescence from Eu^3+^:BaF_2_ fluoride nanocrystals dispersed in sol-gel glass-ceramic materials was successfully observed. The red-to-orange luminescence intensity ratio R/O (Eu^3+^) related to the ^5^D_0_→^7^F_2_ electric-dipole transition (red) and the ^5^D_0_→^7^F_1_ magnetic-dipole transition (orange) was calculated for samples before and after controlled heat-treatment. It was observed a significant decrease of R/O-ratio values from 3.75 (xerogels) to 0.34 (BaF_2_:Eu^3+^ GCs). Moreover, the luminescence lifetimes of the ^5^D_0_ state (Eu^3+^) in glass-ceramic materials containing BaF_2_ nanocrystals were determined (τ_1_ = 2.12 ms, τ_2_ = 4.62 ms) and compared to precursor xerogels (τ = 0.22 ms). The systematic luminescence investigations clearly suggest the successful migration of the optically active Eu^3+^ ions into low-phonon BaF_2_ fluoride nanocrystals distributed within amorphous silica network. According to good luminescence properties of fabricated BaF_2_:Eu^3+^ GCs, we suppose that prepared glass-ceramic material could be consider as a promising candidate to study the efficient energy transfer processes in doubly- (e.g., Tb^3+^/Eu^3+^) or triply-doped (e.g., Gd^3+^/Tb^3+^/Eu^3+^) systems for generation a tunable visible emission.

## Figures and Tables

**Table 1:** The compositions of xerogels and glasses, heat-treatment conditions and sizes on precipitated BaF_2_ nanocrystals in glass-ceramic materials described in current literature.

Xerogels/Glasses Composition (mol %)	Heat-Treatment Conditions		BaF_2_ Nanocrystals Size	Reference
	Temperature	Time		
(95SiO_2_-5BaF_2_):1%Eu^3+ (a)^	320 °C	1 h	3 nm–4 nm	[25]
	540 °C	1 h	3 nm–4 nm	
	800 °C	1 h	7 nm	
68SiO_2_-15BaF_2_-13K_2_CO_3_-2,75La_2_O_3_-1Sb_2_O_3_-0,25Eu_2_O_3_^(b)^	600 °C	24 h	6 nm–10 nm	[26]
	650 °C	24 h	10 nm–20 nm	
(60SiO_2_-20ZnF_2_-20BaF_2_):3%EuF_3_^(b)^	650 °C	2 h	~19 nm	[27]
50SiO_2_-20Al_2_O_3_-18BaF_2_-7NaF-5EuF_3_^(b)^	650 °C	2 h	~40 nm	[28]
(65SiO_2_-14,5B_2_O_3_-11,5Na_2_O-9BaF_2_): 0.1%EuF_3_^(b)^	630 °C	2 h	47 nm	[29]

^(a)^ Materials prepared by sol-gel technique. ^(b)^ Materials prepared by conventional melt-quenching method.
